# AI-Based Sensor Information Fusion for Supporting Deep Supervised Learning

**DOI:** 10.3390/s19061345

**Published:** 2019-03-18

**Authors:** Carson K. Leung, Peter Braun, Alfredo Cuzzocrea

**Affiliations:** 1 Department of Computer Science, University of Manitoba, Winnipeg, MB R3T 2N2, Canada; umbrau73@myumanitoba.ca; 2 Department of Engineering (DIA), University of Trieste, 34127 Trieste, Italy; alfredo.cuzzocrea@dia.units.it

**Keywords:** sensor, information fusion, sensor fusion, artificial intelligence (AI), deep learning, supervised learning, data mining, transportation, geographic information system (GIS), global navigation satellite system (GNSS), global positioning system (GPS)

## Abstract

In recent years, artificial intelligence (AI) and its subarea of deep learning have drawn the attention of many researchers. At the same time, advances in technologies enable the generation or collection of large amounts of valuable data (e.g., sensor data) from various sources in different applications, such as those for the Internet of Things (IoT), which in turn aims towards the development of smart cities. With the availability of sensor data from various sources, sensor information fusion is in demand for effective integration of big data. In this article, we present an AI-based sensor-information fusion system for supporting deep supervised learning of transportation data generated and collected from various types of sensors, including remote sensed imagery for the geographic information system (GIS), accelerometers, as well as sensors for the global navigation satellite system (GNSS) and global positioning system (GPS). The discovered knowledge and information returned from our system provides analysts with a clearer understanding of trajectories or mobility of citizens, which in turn helps to develop better transportation models to achieve the ultimate goal of smarter cities. Evaluation results show the effectiveness and practicality of our AI-based sensor information fusion system for supporting deep supervised learning of big transportation data.

## 1. Introduction

Recent advances in technology have increased the popularity of the area of artificial intelligence (AI) [1,2], which aims to build “intelligent agents” with the ability to correctly interpret external data, learn from these data, and use the learned knowledge for cognitive tasks [3] like reasoning, planning, problem solving, decision making, motion and manipulation. Subareas of AI include robotics, computer vision, natural language processing (NLP), and machine learning [4,5,6,7]. Within the latter, deep learning [8,9,10] has attracted the focus of many researchers. For instance, the development of AlphaGo (which uses deep reinforcement learning) for the board game of Go [11] has drawn the attention of researchers and the general public. In general, deep learning uses deep neural networks (DNNs), convolutional neural networks (CNNs), as well as recurrent neural networks (RNNs) like long short-term memory (LSTM) for supervised, semi-supervised, or unsupervised learning tasks [12,13,14] in various application areas like computer vision and NLP. Recently, deep learning has also been applied to the transportation domain [15,16], but for tasks like traffic flow forecasting, automatic vehicle detection, autonomous driving, and classification of speeding drivers.

Moreover, recent advances in technology have also enabled the generation or collection of large amounts of valuable data from a wide variety of sources in different real-life applications [17,18,19,20,21,22]. For instance, different types of sensor data can be easily generated and collected in various Internet of Things (IoT) [23,24] applications—such as smart homes, smart grids, smart retail, smart cars, and smart cities [25,26]. As an example, sensors (e.g., cameras; digital scanners; light imaging, detection, and ranging (LIDAR) [27]) mounted on aircrafts, small unmanned aerial vehicles (UAVs) [28], and other moving objects such as vehicles [29] have created large volumes of remotely sensed data, geospatial data, spatial-temporal data, and geographic information for the geographic information system (GIS) [30,31]. As another example, sensors on the global navigation satellite system (GNSS) [32]—such as the Global Positioning System (GPS) [33], GLONASS, Gaillieo and Beidou (which are originated in the USA, Russia, the EU and China, respectively), as well as other regional systems—have also created large volumes of geolocation and time information. With big sensory data from these sources and other sensors, sensor information fusion [34,35,36,37]—which integrates sensor data and information from a collection of these heterogeneous or homogeneous sensors to produce accurate, consistent and useful knowledge—is in demand.

In this article, we present an AI-based sensor-information fusion system, which integrates transportation data generated and collected from sensors for the geographic information system (GIS) and global navigation satellite/positioning system (GNSS/GPS). The system also executes deep supervised learning from integrated transportation data to help analysts gain a better understanding of the data and trajectories or mobility of citizens, which in turn helps develop better transportation models to achieve the ultimate goal of smarter cities. Our key contributions include the design of such a sensor-information fusion system to mine and analyze transportation data for supervised learning of these big data sets.

The remainder of this article is organized as follows. The next section presents background materials (e.g., related works). Section 3 describes our method—namely, an AI-based sensor-information fusion system—in detail. Section 4 discusses our evaluation results, and Section 5 draws the conclusions.

## 2. Background Materials and Related Works

In this section, we present some background materials on works related to sensor information fusion and supervised learning of transportation data—specifically, traditional, sensor-based, and sensor fusion-based methods for urban data analytics and machine learning.

### 2.1. Traditional Methods for Urban Data Analytics and Machine Learning

Urban data mining helps discover useful knowledge from urban data, which in turn helps solve some urban issues. For instance, the discovery of popular transportation modes (e.g., bicycles) of residents in a city helps city planners to take appropriate actions (e.g., add more bike lanes). To mine these urban data, researchers have traditionally been using paper-based and telephone-based travel surveys [38]. Unfortunately, these travel surveys can be biased and contain inaccurate data about the movements of their participants. For instance, participants tend to under-report short trips, irregular trips, and car trips. They also tend to over-report public transit trips [39,40].

Alternatively, researchers have also been using commute diaries [41,42], which capture data about people’s daily commutes. Unfortunately, these diaries can be error-prone. When people are asked to use a diary to keep track of their commutes, they often forget to record their commutes throughout the day. When trips are recorded at the end of the day, diary studies can then inherit the same problems as paper-based and telephone-based travel surveys. Moreover, these diaries can also be a mental burden to study the participants, and thus cannot be used long term [43]. Furthermore, as people’s willingness to record trips accurately throughout the day declines with each day of participation, the corresponding accuracy of the commute diaries also drops [44].

### 2.2. Sensor-Based Methods for Urban Data Analytics and Machine Learning

Recent advances in technology have led to the availability of sensor data, which in turn have led to better approaches for urban data mining. To elaborate, sensors enable users to track a large number of movement trajectories that are collected by participants of a study who use GNSS/GPS trackers or other sensors (e.g., accelerometers, barometers, Bluetooth, Wi-Fi, etc.). Hence, these *GPS-based travel surveys* [45,46] are more accurate than the travel surveys and commute diaries. However, the challenge of labeling trip purposes and classifying transportation modes persists. For instance, the manual segmentation of trajectories based on transportation mode can be labor intensive and is likely to be impracticable for big data [47]. Any AI approach to automating such a task would obviously be beneficial to travel studies and other applications (e.g., IoT applications) that rely on contextual knowledge (e.g., the current travel mode of a person). For example, a driver would benefit from receiving a notification from his smartphone or smartwatch about an estimated arrival time for his trip (computed based on his current location, destination, and his interaction or saved frequently visited locations). As another example, urban analysts would benefit from an automatic trip transportation mode labeling method in a way is similar to timeline in Google Maps (which keeps track of a user’s location history and attempts to automatically classify trips with the major transportation mode). However, existing trip transportation mode labeling methods were not very accurate, needed corrections by the user, and do not track when transportation modes were changed. Hence, a more accurate method is needed.

Consider the use of standalone tracking and logging devices, which enables the participants of travel surveys to log sensor data accurately, reliably, and consistently as they have full control over the device and the hardware and software platforms are the same on every device. These devices can log data to local device storages, which are then collected for data retrieval. These devices can also connect to a smartphone application on a participant’s phone via Bluetooth and collect data regularly at intervals for further processing. To a further extent, transportation mode classification could happen on a smartphone, which then could reduce the computational burden on the logger device, decrease both architecture cost (as it requires weaker processing units) and power consumption, and thus increase the battery life. Among related works, Zheng et al. [48] used supervised decision trees and graph-based post-processing after classification to classify transportation modes from GPS data only.

In contrast, Hemminki et al. [49] used only accelerometer data to classify transportation modes (“stationary”, “walk”, “bus”, “tram”, “train”, “metro”). To elaborate, three different classifiers were trained with a combination of AdaBoost and Hidden Markov Model (HMM) for three different classes of modes. Shafique and Hato [50] also used accelerometer data only. They applied multiple machine learning algorithms to perform transportation mode classification and found that the Random Forest algorithm [51] gave accurate classifications.

### 2.3. Sensor Fusion-Based Methods for Urban Data Analytics and Machine Learning

Instead of using only GPS data or only accelerometer data, Ellis et al. [52] applied the Random Forest to both GPS data and accelerometer data for successful transportation mode classification.

Other than using both GPS data and accelerometer data, Hosseinyalamdary et al. [29] used both GIS and GPS data (together with an inertial measurement unit (IMU)). However, they used these data for tracking three-dimensional (3D) moving objects rather than classifying transportation modes. On the hand, Chung and Shalaby [53] developed a system that uses both GPS and GIS data to classify four transportation modes—“walk”, “bicycle”, “bus” and “car”—for GPS-based travel surveys by using a rule-based algorithm and a map-matching algorithm [54] to detect the exact roads people moved on. However, the accuracy of the system is dependent on the corresponding GIS data. Similarly, Stenneth et al. [55] also used both GPS and GIS data when building their real-time transportation mode classification system. To perform the classification, they used the Random Forest as the supervised learning algorithm to identify a person’s current transportation mode.

### 2.4. Summary of Related Works for Urban Data Analytics and Machine Learning

So far, we have described traditional methods for urban data mining, which include paper-based and telephone-based travel surveys [38,39,40], as well as commute diaries [41,42,43,44]. To reduce the human workload and to utilize sensors and AI technologies for automatic processes, GPS-based travel surveys [45,46,47] were used. In recent years, advances in technologies have enabled the use of some combinations of data from different sensors (e.g., GNSS/GPS, accelerometers) and other modern smartphone sensors (e.g., barometer, magnetometer, etc.). Some related works [48] use only GPS data, while some others [49,50] use only accelerometer data. In addition, some related works [52] integrate both GPS and accelerometer data (i.e., an example of sensor information fusion), while some others [53,54,55] integrate both GPS and GIS data (i.e., another example of sensor information fusion). However, to the best of our knowledge, none of them combines GNSS/GPS, accelerometer, and GIS data in a single system. In contrast, our system integrates GNSS/GPS, accelerometer, and GIS data for urban data analytics and machine learning.

## 3. Our Methods

In this section, we describe our system for both sensor information fusion (of GNSS/GPS data, accelerometer data, and GIS data) and deep supervised learning (of these transportation data to classify ground transportation mode). The system consists of two modules:

- The big data management module, and
- the big data analytics module.

### 3.1. The Big Data Management Module of Our System

Our big data management module collects, integrates, and manages a wide variety of data—namely, GNSS/GPS, accelerometer, and GIS data—from multiple data sources to produce consistent and useful information so as to facilitate sensor information fusion. Specifically, our module collects and integrates the following data:

- Trip traces (GNSS/GPS locations),
- trip accelerometer data, and
- GIS information.

Note that our module collects and integrates both trip traces (GNSS/GPS locations) and trip accelerometer data and stores them in a database for big data (e.g., MongoDB). To generate and collect data for a new trip or trip leg, users simply use an application (app) on their smartphones. Then, the users choose their transportation modes (e.g., “walk”, “run”, “bicycle”, motorcycle”, “car”, “bus”) from a pop-up list at the beginning of a trip and when they transit between different modes during a trip. Moreover, the users can view trip information (e.g., their saved trip log with speed information, alerts, and map). In addition, the app also keeps track of users’ movement. It allows users to manage their recorded trips, start new trip recordings, and upload existing trips. By default, the GNSS/GPS sampling rate was set to 1 Hz and the accelerometer sampling was at 22 Hz. However, users have the freedom to modify these setting values.

Knowing the difficulty in obtaining a complete set of GIS information in some real-life situations, our module only requires GIS information in the form of bus stop locations in a city. The module collects this GIS information via its transit application programming interface (API) when “bus” is one of the ground transportation modes for classification and stores the information as a vector file in JavaScript Object Notation (JSON) format.

### 3.2. Big Data Analytics Module of Our System

Our big data analytics module facilitates sensor information fusion, analyzes the input data, and turn them into useful knowledge. Specifically, our module first segments every trip (which is simply a collection of data points collected during a person’s entire commute from origin to destination—say, from home to work) based on the transportation mode used in each segment. For example, for a trip from home to work can be divided into the following five segments:

- Walking from home to the departure bus stop,
- busing from the departure bus stop to an immediate bus station,
- walking from the bus station to its nearest metro station,
- taking the metro from the metro station to the destination metro stop, and
- walking from the destination metro stop to the office.

To segment a trip, its associated data are divided into many small windows of equal-time intervals. When a transportation mode change occurs, data are assigned to different windows so that no two transportation modes are mixed within the same window. Segmenting the data into small windows means that they can be classified in real-time. For instance, as soon as a sufficient amount of data has been collected to fill a new window, the window can be classified with a transportation mode. Once every window is classified with a transportation mode, the user simply concatenates the windows/trip segments (each of which is labeled with a transportation mode) and presents each label on a map with a color-scheme for different transportation modes. Once the trip is rendered on a map, the user can easily identify different legs of the trip by simply looking at the different colors of the trip.

After segmenting the trip, our big data analytics module then extracts appropriate features for transportation mode classification. Specifically, it extracts the following three key types of features: GNSS/GPS-based, accelerometer-based, and GIS-based features from the integrated sensor information. Among them, GNSS/GPS-based features capture the following geo-location and time information provided by GNSS/GPS sensors:

- Average speed (by default, measured in km/h; can be also measured in mph);
- maximum speed (by default, measured in km/h; can be also measured in mph);
- minimum altitude (by default, measured in m; can be also measured in ft);
- average altitude (by default, measured in m; can be also measured in ft);
- maximum altitude (by default, measured in m; can be also measured in ft);
- minimum location accuracy (by default, measured in m; can be also measured in ft);
- average location accuracy (by default, measured in m; can be also measured in ft);
- maximum location accuracy (by default, measured in m; can be also measured in ft);
- travel distance (by default, measured in m; can be also measured in ft), which computes haversine distance d between two points *p*_1_ and *p*_2_ on a sphere as follows:
  (1) $$d=2r\sin^{-1}\left(\sqrt{\sin^{2}\left(\frac{lat_{2}-lat_{1}}{2}\right)+\cos\left(lat_{1}\right)\cos\left(lat_{2}\right)\sin^{2}\left(\frac{long_{2}-long_{1}}{2}\right)}\right)$$
  where (i) *r* is the radius of the spherical Earth, (ii) *long*_1_ and *lat*_1_ are respectively the longitude and latitude of *p*_1_, and (iii) *long*_2_ and *lat*_2_ are respectively the longitude and latitude of *p*_2_; and
- a Boolean feature indicating whether there is GNSS/GPS signal or no signal.

To complement these GNSS/GPS-based features, accelerometer-based features capture the following measurement on the acceleration of the sensed movement of different transportation modes (e.g., automobile):

- Minimum magnitude;
- 25th percentile magnitude, which captures the average of all magnitude in the 25th percentile;
- average magnitude;
- 75th percentile magnitude, which captures the average of all magnitude in the 75th percentile;
- maximum magnitude;
- standard deviation for magnitude;
- lag 1 autocorrelation;
- correlation between x- and y-axes;
- correlation between x- and z-axes;
- correlation between y- and z-axes;
- average roll, which captures the average “bank angle” about rotations along the x-axis (i.e., longitudinal axis);
- average pitch, which captures the average “elevation” about rotations along the y-axis (i.e., lateral or transverse axis); and
- average yaw, which captures the average “bearing” about rotations along the z-axis (i.e., vertical axis).

To complement both the aforementioned GNSS/GPS-based features and these accelerometer-based features, GIS-based features capture the following geographic information provided by sensors:

- The number of bus stops, which captures the number of unique bus stops within the window;
- the number of stops at (or near) bus stops, which captures the number of stops near all the bus stops within the window;
- distance to the closest bus stop (by default, measured in m; can be also measured in ft), which captures the distance to a bus stop that is closest to the person within the entire window; and
- a Boolean feature indicating whether or not a person stopped at any of the nearby bus stops within the window.

After extracting and integrating all these GNSS/GPS-based, accelerometer-based, and GIS-based features (i.e., sensor information fusion), our module then builds, trains, and validates a predictive analytic model for the classification of transportation modes. Specifically, it builds a deep random forest classifier. This classifier is an ensemble deep-learning method for supervised learning of the extracted features to classify transportation modes. We first use multiple random subsets of the extracted features, which are stored on a per-trip basis, to construct and train multitudes of random decision trees within the random forest. We then select the best (or the most popular) classified label for classification and prediction. For each trip, there is a set of feature windows. Our module shuffles trips for each transportation mode and then splits them into two sets: (i) the training set, and (ii) the testing/validation set. By default, our module is set to use 70% of the data for stratified 10-fold cross-validation with a 50/50 partition split between the training and the testing data. However, users have the freedom to modify these setting values. After building and training the classifier, our big data analytics module classifies unseen data according to the ground transportation mode (e.g., “walk”, “bicycle”, “bus”, “car”) used by the user. It then returns the classified trips back to our big data management module for storage in the databases for big data (e.g., MongoDB).

## 4. Results and Discussion

This section shows and discusses our evaluation results on the presented supervised learning system when classifying and predicting ground transportation mode. We conduct our evaluation on real transportation data on a computer running Ubuntu 16.04 LTS as the main operating system, an AMD Phenom II X6 1100T CPU with 6 cores clocked at 3.3–3.7 GHz as the CPU, 16 GB of RAM, and a solid-state drive. We collected the GIS information (e.g., 5170 bus stop locations in Winnipeg) from Winnipeg Transit’s Open Data Web Service by querying trip data via the Winnipeg Transit API. The GIS information is stored as a vector file in JSON format. Users anonymously and securely uploaded their saved accelerometer-based and GNSS/GPS-based data via the mobile applications or dashboard. The trip information from users was then stored in MongoDB, which supports basic geospatial query capabilities. The trip information was collected throughout a year, which contains trips with different weather and road conditions from summer to winter times, including humid and hot summer days with temperatures around +30 °C, to mild autumn days with temperatures ideal for walking and cycling, to windy and cold winter days with temperature around −30 °C. It also includes dry, wet, foggy, slippery, icy, snowy, and blizzard road conditions. It captures the ground transportation modes (e.g., “walk”, “car”, “bus”) used by the user at the time of commute.

### 4.1. Evaluation of the Window Size for Trip Segmentation

**Figure 1:**
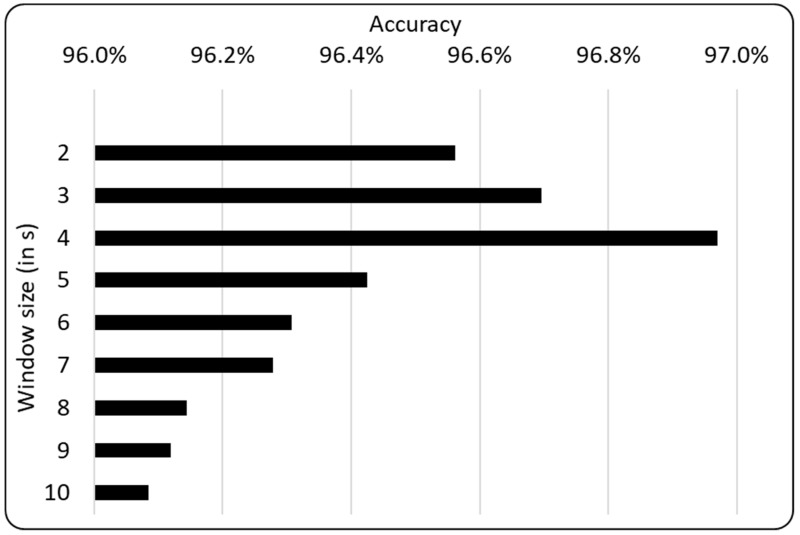
Evaluation results on window size vs. accuracy.

Recall from Section 3.2 that our big data analytics module segments a trip into many small windows of equal time intervals. Our first set of evaluations determines an appropriate window size. We varied the window size from intervals of two seconds (2 s) to intervals of ten seconds (10 s). The evaluation results shown in Figure 1 reveal that, when the window size increased from an interval of 2 s to an interval of 4 s, the accuracy increased. However, when the window size increased from an interval of 4 s to an interval of 10 s, the accuracy decreased. Consequently, a window size of 4 s gave the most accurate classification. A reason why a window size of 4 s gave a good fit is that, when the window size increased from an interval of 4 s to an interval of 10 s, the window became bigger in such a way that it became less adequate in capturing the underlying transportation modes or changes of modes, thus leading towards under-fitting. This explains why the corresponding accuracy decreased when the window size increased beyond 4 s.

### 4.2. Evaluation of the Accuracy of Our System when Compared with Related Works

Recall from Section 2.4 that some related works [48] use only GPS data, while some [49,50] use only accelerometer data. In addition, some related works [52] integrate both GPS and accelerometer data, while some [53,54,55] integrate both GPS and GIS data. However, to the best of our knowledge, none of them combines GNSS/GPS, accelerometer and GIS data in a single system. In contrast, our system integrates GNSS/GPS, accelerometer, and GIS data for urban data mining. Our second set of evaluations involves comparing the effectiveness of our system with related works. To evaluate the effectiveness of a predictive analytics system, we use the standard measures of precision, recall and accuracy as measurements. Let:

- TP denote the true positive (i.e., when the system correctly predicts the positive class of transportation mode),
- TN denote the true negative (i.e., when the system correctly predicts the negative class of transportation mode),
- FP denote the false positive (i.e., when the system incorrectly predicts the positive class of transportation mode), and
- FN denote the false negative (i.e., when the system incorrectly predicts the negative class of transportation mode).

Precision measures the positive predictive/classified value, i.e., the fraction of true positives among all positives (i.e., true and false positives):

Precision = TP/(TP + FP)(2)

Recall measures the true positive rate or sensitivity, i.e., the fraction of true positives among true positives and false negatives:

Recall = TP/(TP + FN)(3)

Accuracy measures the fraction of true positives and true negatives among all predications/classifications (i.e., among all positives and negatives):

Accuracy = (TP + TN)/(TP + TN + FP + TN)(4)

With these metrics, we measured the accuracy of the prediction when training the classifier on a fusion of all three types of GNSS/GPS-based, accelerometer-based, and GIS-based sensor data. We compared this with the accuracy of the prediction when training the classifier on only GNSS/GPS-based data or only accelerometer-based data. We also compared it with the accuracy of the prediction when training the classifier on the following combinations:

- Both GNSS/GPS-based and accelerometer-based data, or
- both GNSS/GPS-based and GIS-based data.

**Figure 2:**
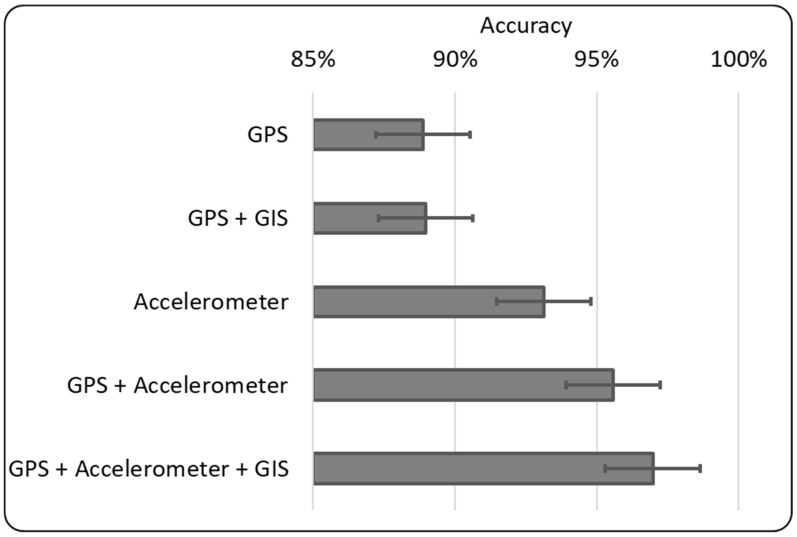
Evaluation results on different combinations of sensor data vs. accuracy.

The evaluation results shown in Figure 2 reveal that among the five combinations (four combinations in related works and our sensor information fusion), using only GNSS/GPS-based data led to the lowest accuracy. Integration of GIS-based data into the GNSS/GPS-based data improved accuracy. Moreover, the use of only accelerometer-based data led to higher accuracy. Integration of GNSS/GPS-based data into the accelerometer-based data further improved accuracy. Fusion of all GNSS/GPS-based data, GIS-based data, and accelerometer-based data led to the highest accuracy.

The results on the five combinations of sensor data are consistent with the comparisons of our system with the following related works:

- Recall from Section 2.2 that Zheng et al. [48] applied decision trees (cf. our random forest classifier) to only GPS-based data, which gave a high accuracy (of above 85%).
- Recall from Section 2.3 that Stenneth et al. [55] applied the Random Forest to both GPS-based and GIS-based data, which led to slightly higher accuracy than the use of only GPS-based data.
- Recall from Section 2.2 that Shafique and Hato [50] found that their application of Random Forest to only accelerometer-based data gave accurate classification, with an improvement in accuracy of about 5% over the two aforementioned related works on using either only GPS-based data or (GPS + GIS)-based data.
- Recall from Section 2.3 that Ellis et al. [52] also applied the Random Forest but to both GPS-based and accelerometer-based data, which lead to an improvement of another 2% in accuracy over the use of solely accelerometer-based data.

Our system, which uses GIS-based data in addition to both GNSS/GPS-based and accelerometer-based data, led to further improvement in accuracy over the last/fourth related work that uses (GPS + accelerometer)-based data. When compared the first two related works that use only GPS-based data and (GPS + GIS)-based data, our system provides nearly a 10% improvement in accuracy.

## 5. Conclusions

In this article, we present an AI-based sensor-information fusion system for supporting deep supervised learning of transportation data generated, collected and integrated from various types of sensors. These include geo-location and time information provided by GNSS/GPS sensors, accelerometer data regarding the sensed movement of some modes of transportation, and sensor data from the geographic information system (GIS). This is a fusion of GNSS/GPS-based, accelerometer-based, and GIS-based sensor information. Our system consists of two modules: One for big data management of sensor data, and another for big data analytics of the sensor data. Specifically, our deep random forest classifier in the latter of the two modules performs supervised learning on the sensor data. Evaluation results show that our combination or fusion of all three types of data—namely, GNSS/GPS-based, accelerometer-based and GIS-based sensor data—led to more accurate prediction and classification of the ground transportation mode, which in turn helps urban data miners, city planners, and related professionals to achieve the goal of smart cities.

Recall from Section 3.2 that we captured the travel distance by using haversine distance, which determines the great-circle distances between two points on a sphere. Given that the Earth is not a perfect sphere, as ongoing work, we are exploring other distance metrics such as Vincenty’s formulae for computing geodesic distance. Moreover, as our fusion of sensor information leads to more accurate predictive analytics results, a logical future research direction is to exploit additional useful features within the three existing types of sensor data. A related future research direction is to explore additional types of sensor data beyond the three existing types.

## References

[1] Guo K., Lu Y., Gao H., Cao R. (2018). Artificial intelligence-based semantic Internet of Things in a user-centric smart city. Sensors.

[2] Sandino J., Pegg G., Gonzalez L.F., Smith G. (2018). Aerial mapping of forests affected by pathogens using UAVs, hyperspectral sensors, and artificial intelligence. Sensors.

[3] Deng D., Leung C.K., Wodi B.H., Yu J., Zhang H., Cuzzocrea A. (2018). An innovative framework for supporting cognitive-based big data analytics for frequent pattern mining. Proceedings of the IEEE ICCC 2018.

[4] Brown J.A., Cuzzocrea A., Kresta M., Kristjanson K.D.L., Leung C.K., Tebinka T.W. (2017). A machine learning system for supporting advanced knowledge discovery from chess game data. Proceedings of the IEEE ICMLA 2017.

[5] Leung C.K., MacKinnon R.K., Wang Y. (2014). A machine learning approach for stock price prediction. Proceedings of the IDEAS 2014.

[6] Morris K.J., Egan S.D., Linsangan J.L., Leung C.K., Cuzzocrea A., Hoi C.S. (2018). Token-based adaptive time-series prediction by ensembling linear and non-linear estimators: A machine learning approach for predictive analytics on big stock data. Proceedings of the IEEE ICMLA 2018.

[7] Zhang L., Xiao N., Yang W., Li J. (2019). Advanced heterogeneous feature fusion machine learning models and algorithms for improving indoor localization. Sensors.

[8] Islam M., Sohaib M., Kim J., Kim J. (2018). Crack classification of a pressure vessel using feature selection and deep learning methods. Sensors.

[9] Xiao L., Zhang Y., Peng G. (2018). Landslide susceptibility assessment using integrated deep learning algorithm along the China-Nepal Highway. Sensors.

[10] Strauß S. (2018). From big data to deep learning: A leap towards strong AI or ‘intelligentia obscura’?. Big Data Cogn. Comput..

[11] Leung C.K., Kanke F., Cuzzocrea A. (2018). Data analytics on the board game Go for the discovery of interesting sequences of moves in joseki. Procedia Comput. Sci..

[12] Castagno J., Atkins E. (2018). Roof shape classification from LiDAR and satellite image data fusion using supervised learning. Sensors.

[13] Li M., Li Q., Liu G., Zhang C. (2018). Generative adversarial networks-based semi-supervised automatic modulation recognition for cognitive radio networks. Sensors.

[14] Wang J., Sanchez J.A., Ayesta I., Iturrioz J.A. (2018). Unsupervised machine learning for advanced tolerance monitoring of wire electrical discharge machining of disc turbine fir-tree slots. Sensors.

[15] Bhavsar P., Safro I., Bouaynaya N., Polikar R., Dera D. (2017). Machine learning in transportation data analytics. Data Analytics for Intelligent Transportation Systems.

[16] Nguyen H., Kieu L., Wen T., Cai C. (2018). Deep learning methods in transportation domain: A review. IET Intell. Transp. Syst..

[17] Braun P., Cameron J.J., Cuzzocrea A., Jiang F., Leung C.K. (2014). Effectively and efficiently mining frequent patterns from dense graph streams on disk. Procedia Comput. Sci..

[18] Jiang F., Leung C.K. (2015). A data analytic algorithm for managing, querying, and processing uncertain big data in cloud environments. Algorithms.

[19] Lakshmanan L.V.S., Leung C.K., Ng R.T. (2000). The segment support map: Scalable mining of frequent itemsets. ACM SIGKDD Explor..

[20] Leung C.K. (2018). Frequent itemset mining with constraints. Encyclopedia of Database Systems.

[21] Li K.C., Jiang H., Yang L.T., Cuzzocrea A. (2015). Big data: Algorithms, analytics, and applications.

[22] Wu Z., Yin W., Cao J., Xu G., Cuzzocrea A. (2013). Community detection in multi-relational social networks. Proceedings of the WISE 2013.

[23] Braun P., Cuzzocrea A., Leung C.K., Pazdor A.G.M., Tanbeer S.K., Grasso G.M. (2018). An innovative framework for supporting frequent pattern mining problems in IoT environments. Proceedings of the ICCSA 2018, Part V.

[24] Drenoyanis A., Raad R., Wady I., Krogh C. (2019). Implementation of an IoT based radar sensor network for wastewater management. Sensors.

[25] Leung C.K., Braun P., Pazdor A.G.M. (2018). Effective classification of ground transportation modes for urban data mining in smart cities. Proceedings of the DaWaK 2018.

[26] Morales Lucas C., de Mingo López L.F., Gómez Blas N. (2018). Natural computing applied to the underground system: A synergistic approach for smart cities. Sensors.

[27] Wang C., Ji M., Wang J., Wen W., Li T., Sun Y. (2019). An improved DBSCAN method for LiDAR data segmentation with automatic Eps estimation. Sensors.

[28] Popescu D., Dragana C., Stoican F., Ichim L., Stamatescu G. (2018). A collaborative UAV-WSN network for monitoring large areas. Sensors.

[29] Hosseinyalamdary S., Balazadegan Y., Toth C. (2015). Tracking 3D moving objects based on GPS/IMU navigation solution, laser scanner point cloud and GIS data. ISPRS Int. J. Geo-Inf..

[30] Ait Lamqadem A., Pradhan B., Saber H., Rahimi A. (2018). Desertification sensitivity analysis using MEDALUS model and GIS: A case study of the oases of Middle Draa Valley, Morocco. Sensors.

[31] Burrough P.A., McDonnell R.A., Lloyd C.D. (2015). Principles of Geographical Information Systems.

[32] Robustelli U., Baiocchi V., Pugliano G. (2019). Assessment of dual frequency GNSS observations from a Xiaomi Mi 8 Android smartphone and positioning performance analysis. Electronics.

[33] Zimmermann F., Schmitz B., Klingbeil L., Kuhlmann H. (2019). GPS multipath analysis using Fresnel zones. Sensors.

[34] Choi S., Cho S. (2018). Sensor information fusion by integrated AI to control public emotion in a cyber-physical environment. Sensors.

[35] de la Iglesia D.H., Villarrubia G., de Paz J.F., Bajo J. (2017). Multi-sensor information fusion for optimizing electric bicycle routes using a swarm intelligence algorithm. Sensors.

[36] Jing L., Wang T., Zhao M., Wang P. (2017). An adaptive multi-sensor data fusion method based on deep convolutional neural networks for fault diagnosis of planetary gearbox. Sensors.

[37] Kim H., Suh D. (2018). Hybrid particle swarm optimization for multi-sensor data fusion. Sensors.

[38] Murakami E., Wagner D.P., Neumeister D.M. (2000). Using global positioning systems and personal digital assistants for personal travel surveys in the United States. Proceedings of the International Conference on Transport Survey Quality and Innovation 1997.

[39] Ettema D., Timmermans H., van Veghel L. (1997). Effects of Data Collection Methods in Travel and Activity Research.

[40] Stopher P.R. (1995). Household travel surveys: Cutting-edge concepts for the next century. Proceedings of the Conference on Household Travel Surveys 1995.

[41] Maat K., Timmermans H.J.P., Molin E. (2004). A model of spatial structure, activity participation and travel behavior. Proceedings of the WCTR 2004.

[42] Stopher P.R. (1992). Use of an activity-based diary to collect household travel data. Transportation.

[43] Schlich R., Axhausen K.W. (2003). Habitual travel behaviour: Evidence from a six-week travel diary. Transportation.

[44] Arentze T., Dijst M., Dugundji E., Joh C., Kapoen L., Krygsman S., Maat K., Timmermans H. (2001). New activity diary format: Design and limited empirical evidence. Transp. Res. Rec..

[45] Forrest T., Pearson D. (2005). Comparison of trip determination methods in household travel surveys enhanced by a global positioning system. Transp. Res. Rec..

[46] Wolf J., Guensler R., Bachman W. (2001). Elimination of the travel diary: Experiment to derive trip purpose from global positioning system travel data. Transp. Res. Rec..

[47] Biljecki F., Ledoux H., van Oosterom P. (2013). Transportation mode-based segmentation and classification of movement trajectories. Int. J. Geogr. Inf. Sci..

[48] Zheng Y., Chen Y., Li Q., Xie X., Ma W. (2010). Understanding transportation modes based on GPS data for web applications. ACM Trans. Web (TWEB).

[49] Hemminki S., Nurmi P., Tarkoma S. (2013). Accelerometer-based transportation mode detection on smartphones. Proceedings of the ACM SenSys 2013.

[50] Shafique M.A., Hato E. (2015). Use of acceleration data for transportation mode prediction. Transportation.

[51] Breiman L. (2001). Random forests. Mach. Learn..

[52] Ellis K., Godbole S., Marshall S., Lanckriet G., Staudenmayer J., Kerr J. (2014). Identifying active travel behaviors in challenging environments using GPS, accelerometers, and machine learning algorithms. Front. Public Health.

[53] Chung E., Shalaby A. (2005). A trip reconstruction tool for GPS-based personal travel surveys. Transp. Plan. Technol..

[54] Greenfeld J. (2002). Matching GPS observations to locations on a digital map. Proceedings of the Transportation Research Board 81st Annual Meeting.

[55] Stenneth L., Wolfson O., Yu P.S., Xu B. (2011). Transportation mode detection using mobile phones and GIS information. Proceedings of the ACM SIGSPATIAL GIS 2011.

